# Self-assembled honeycomb lattice in the monolayer of cyclic thiazyl diradical BDTDA (= 4,4′-bis(1,2,3,5-dithiadiazolyl)) on Cu(111) with a zero-bias tunneling spectra anomaly

**DOI:** 10.1038/srep18359

**Published:** 2015-12-17

**Authors:** Masayuki Yamamoto, Rie Suizu, Sudipta Dutta, Puneet Mishra, Tomonobu Nakayama, Kazuyuki Sakamoto, Katsunori Wakabayashi, Takashi Uchihashi, Kunio Awaga

**Affiliations:** 1 Department of Nanomaterials Science, Graduate School of Advanced Integration Science, Chiba University, 1-33 Yayoi-cho, Inage-ku, Chiba 263-8522, Japan; 2 International Center for Materials Nanoarchitectonics, National Institute for Materials Science, 1-1 Namiki, Tsukuba 305-0044, Japan; 3 Department of Chemistry and Research Center for Materials Science, Nagoya University, Furo-cho, Chikusa-ku, Nagoya 464-8602, Japan; 4 CREST, JST, 4-1-8 Honcho, Kawaguchi 332-0012, Japan

## Abstract

Scanning tunneling microscopy (STM) observation reveals that a cyclic thiazyl diradical, BDTDA (= 4,4′-bis(1,2,3,5-dithiadiazolyl)), forms a well-ordered monolayer honeycomb lattice consisting of paramagnetic corners with unpaired electrons on a clean Cu(111) surface. This BDTDA lattice is commensurate with the triangular lattice of Cu(111), with the former being 3 × 3 larger than the latter. The formation of the BDTDA monolayer structure, which is significantly different from its bulk form, is attributed to an interaction with the metal surface as well as the intermolecular assembling forces. STM spectroscopy measurements on the BDTDA molecules indicate the presence of a characteristic zero-bias anomaly centered at the Fermi energy. The origin of this zero-bias anomaly is discussed in terms of the Dirac cones inherent to the honeycomb structure.

Recently, highly-ordered molecular self-assembled monolayers at solid interfaces have attracted much attention in organic electronics and spintronics^1^,^2^,^3^,^4^,^5^. Indeed, such assembly can be a new stage for organic molecules to demonstrate novel properties and functions arising from the interactions with substrate and/or the formations of 2D architectures that are different from those of bulk crystals. Of the 2D architectures, those forming honeycomb lattice would have particular interest due to the possibility of showing exotic electronic structure such as the Dirac cone, like the electronic structure of graphene^6^,^7^ that also has a honeycomb structure. In this perspective, it is interesting to investigate monolayer structures formed by organic molecules with strong self-assembling forces, instead of those that are weakly connected with each other by van der Waals interactions or hydrogen bonding like^1^,^2^,^3^,^4^,^5^, since a relatively strong intermolecular interaction is necessary for the formation of fascinating 2D electronic structures.

Cyclic thiazyl radicals are chemically-stable open-shell molecules and their solid-state properties have been studied extensively as building blocks of conductors, magnets, and optical materials^8^,^9^,^10^. In solid states, radical molecules always exhibit multidimensional networks due to the combination of the exchange interactions between the radical moieties and the electrostatic interactions caused by the electrically-polarized S-N bonds^8^,^9^,^10^. The strongly-correlated electron systems in the thiazyl radicals have provided various physical properties and functions such as drastic phase transitions, magnetic orderings, non-linear transport, and photo-induced phenomena^10^.

The present compound, BDTDA (= 4,4-bis(1,2,3,5-dithiadiazolyl)^11^,^12^, see Fig. 1(a)), is a so-called disjoint diradical^13^,^14^,^15^ in the thiazyl radical family, in which the two unpaired electrons are localized on separate five-membered rings and the exchange interactions between the two radical centers are very small^12^. Highly-oriented BDTDA thick film has been reported to show an anomalous transient photocurrent that is induced by an effective charge separation at the interface between itself and an electrode^16^,^17^,^18^,^19^. However, although BDTDA monolayer films may also have the potential for exotic 2D physical properties, there has been no study on such system so far. In this present work, we performed scanning tunneling microscopy (STM) and spectroscopy (STS) measurements of the molecular arrangement and electronic structure of a BDTDA monolayer film formed on a clean Cu(111) surface, and discuss its amazing property based on density functional theory (DFT) calculation.

## Results and Discussions

**Figure 1:**
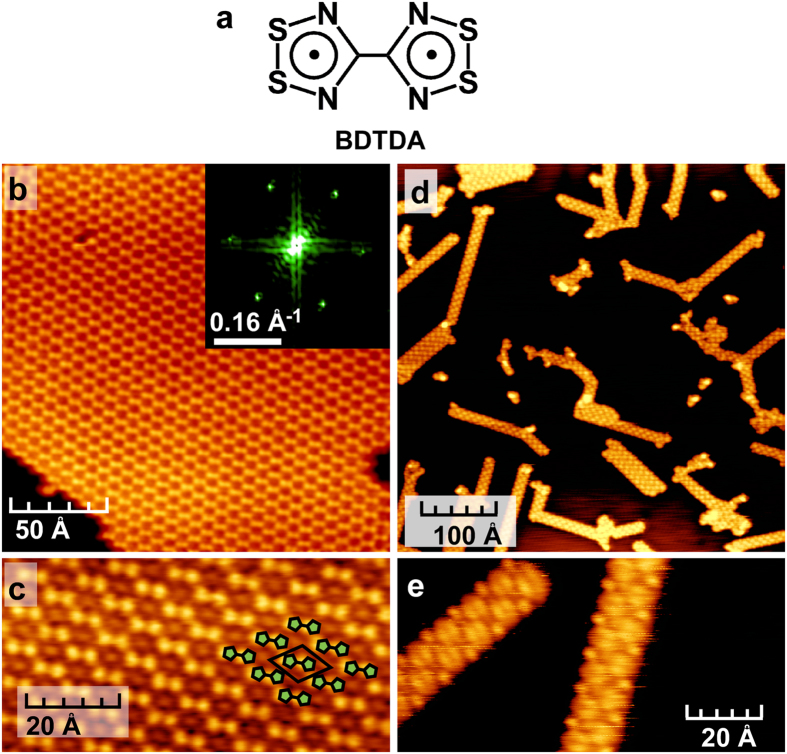
The formation of the 2D BDTDA honeycomb structure on Cu(111) and the 1D nanoribbon on Ag(111). (**a**) Molecular structure of BDTDA. The dots in the pentagons represent localized unpaired electrons. (**b**,**c**) Topography of the 2D BDTDA honeycomb structure on Cu(111); (**b**) 200 × 200 Å^2^, 100 pA, −0.8 V, 80 K. The inset shows the Fast Fourier Transform (FFT) image. (**c**) 80 × 40 Å^2^, 50 pA, −0.8 V, 80 K. (**d**,**e**) Topography of the 1D BDTDA nanoribbons on Ag(111); (**d**) 500 × 500 Å^2^, 100 pA, −2 V, 80 K. (**e**) 100 × 50 Å^2^, 140 pA, −0.1 V, 80 K.

**Figure 2:**
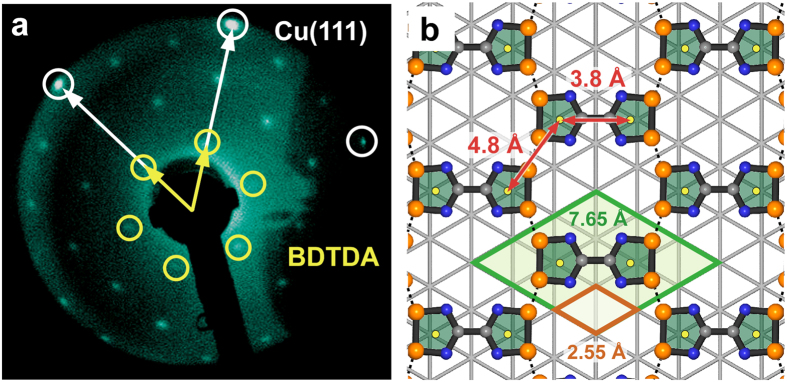
The relation between the BDTDA monolayer and Cu(111) surface. (**a**) LEED pattern of BDTDA/Cu(111). (**b**) Expected molecular arrangement of BDTDA on the Cu(111). Sulfur, nitrogen, and carbon atoms are represented by the orange, navy, and gray spheres, respectively. The substrate is represented by the light gray triangle grid. The orange and green rhomboids are the unit cells of the Cu(111) and BDTDA honeycomb structure, respectively. The yellow dots are the centroids of the pentagonal 1,2,3,5-dithiadiazolyl group.

The STM measurements for the BDTDA monolayers on Cu(111) were performed in a temperature range between 8 K and room temperature. Figure 1(b,c) show the STM images at 80 K for scan areas of 200 × 200 and 80 × 40 Å^2^, respectively. These images show the 2D honeycomb monolayer structure of BDTDA, in which the molecules are structurally well arranged without notable defects. The Fast Fourier Transform (FFT) of the STM image shown in the inset of Fig. 1(b) supports the formation of a large hexagonal net. We note that there was no structural transition of the BDTDA honeycomb lattice at any of the measurement temperatures. The interval between the dots within the bright binary dots in Fig. 1(c) is 3.7 Å, and this value is comparable to the intramolecular distance between the pentagon centers in BDTDA in bulk crystals (3.80 Å)^11^. We therefore attribute each bright binary dot to one individual BDTDA molecule, and consider that the honeycomb structure is formed by short contacts between the radical moieties, as displayed in Fig. 2(b). Since each corner of this honeycomb lattice consists of a radical moiety and thus has one electron, this structure can be regarded as “molecular graphene”^20^,^21^. So far, the formation of molecule-based 2D honeycomb networks has been reported in closed-shell compounds only^1^,^2^,^3^,^4^,^5^, and the BDTDA lattice on Cu(111) is the first example of a honeycomb lattice formed by paramagnetic corners with unpaired electrons. This BDTDA honeycomb lattice on Cu(111) is completely different from the bulk crystal structure of BDTDA^11^, which consists of an alternating 1D π-stacking along the monoclinic *a* axis and a herringbone-type 2D network parallel to the *bc* plane, as shown in Figure S1 (see Supplementary Information). In this herringbone-type 2D network, the molecular planes are parallel to the *bc* plane and are connected by side-by-side S···S and S···N short contacts. This bulk structure is maintained even in a BDTDA film with a thickness of 100 nm^16^. These facts indicate that interaction with the metal surface as well as the intermolecular assembling forces is the key for formation of a honeycomb monolayer on Cu(111). In fact, the selection of the substrate is quite important; BDTDA forms only a 1D nanoribbon structure on Ag(111) and not a 2D network, as shown in Fig. 1(d,e). The lattice constant of Ag is approximately 13% longer than that of Cu, and this difference will make the BDTDA honeycomb lattice incommensurate on a Ag(111) surface. We therefore conclude that the difference in lattice constant greatly affects the structure of BDTDA networks.

To examine the relation between the monolayer of BDTDA and the Cu(111) surface, LEED measurements were performed on BDTDA/Cu(111). In the LEED image shown in Fig. 2(a), the bright spots close to the screen fringe (open white circles) are assigned to a diffraction from the underlying Cu(111) lattice and the others (open yellow circles) are considered to derive from the honeycomb lattice of BDTDA. It is clearly seen that the honeycomb lattice of BDTDA is commensurate with the triangular lattice of Cu(111); the former is 3 × 3 larger than the latter. Considering the lattice constant of Cu(111), which is 2.55 Å, that of the BDTDA monolayer becomes 7.65 Å. In Fig. 1(c), all bright binary dots show uniform brightness, indicating that the molecular plane of BDTDA is parallel to the Cu(111) surface. Taking the LEED results into account, the molecular arrangement would be like that shown in Fig. 2(b). Note that this figure only shows the molecular arrangement of the BDTDA honeycomb network and that of the Cu(111) substrate irrelevantly, since the registry of the network with the surface is unclear, and the molecular coordinates of BDTDA are extracted from those in bulk crystal. In this case, the intermolecular distance between the pentagon centers becomes 4.8 Å, a value that is consistent with the interval between the inter-binary dots (4.6–4.9 Å) in Fig. 1(c). However, this distance of 4.8 Å is much shorter than the corresponding distances in the bulk crystal (5.3 and 6.0 Å)^11^, and thus strongly indicates the presence of a strong intermolecular interaction between the pentagonal 1,2,3,5-dithiadiazolyl groups.

**Figure 3:**
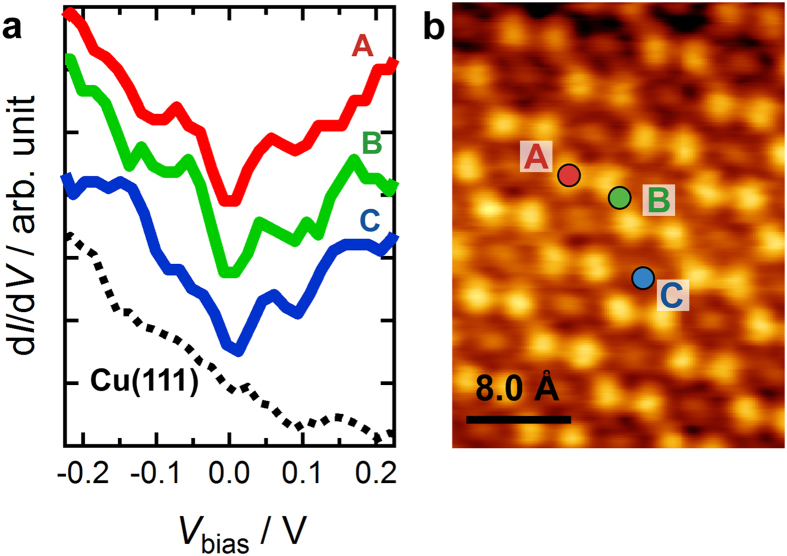
The zero-bias anomaly centered at the Fermi energy in the d*I*/d*V* spectra. (**a**) A series of d*I*/d*V* spectra of the BDTDA honeycomb lattice on Cu(111) in the voltage range from −0.2 to 0.2 V, taken by keeping the tip over the positions marked by (A–C) in panel (**b**). The dotted curve shows the spectrum obtained using a clean Cu(111) substrate. (**b**) The tip positions for STS measurements: on a binary bright dot (A), a node between binary bright dots (B), and the center of a hexagon (C).

STS measurements were carried out to reveal the electronic structure of the honeycomb lattice of BDTDA on Cu(111). The differential conductance (d*I/*d*V)* spectra were acquired using the standard lock-in ac detection, where a modulation voltage (5–20 mV_p-p_ with a frequency of 477 Hz) was superimposed to the sample bias voltage with the feedback loop open. Figure 3(a) shows the d*I*/d*V* spectra of BDTDA/Cu(111) taken by keeping the tip over the positions marked by **A**–**C** in Fig. 3(b). The zero-bias anomalies, namely V-shape spectra with their minimums at the Fermi energy, are observed at all measuring positions. Since the spectra are drastically different from that of Cu(111) (see the dotted line in Fig. 3(a)), the origin of the V-shape dip structure observed in the d*I/*d*V* spectra is attributed to the electronic states of BDTDA molecules. In order to exclude the possibility of a tip artifact effect, we performed STS measurements on a clean Cu(111) substrate just after the d*I*/d*V* measurements for the BDTDA honeycomb lattice (Figure S3(a) of Supplementary Information). The d*I*/d*V* spectra of the clean surface clearly indicate the presence of the surface state of Cu(111) at −0.45 eV^22^,^23^ and therefore the absence of molecular contamination on the tip.

**Figure 4:**
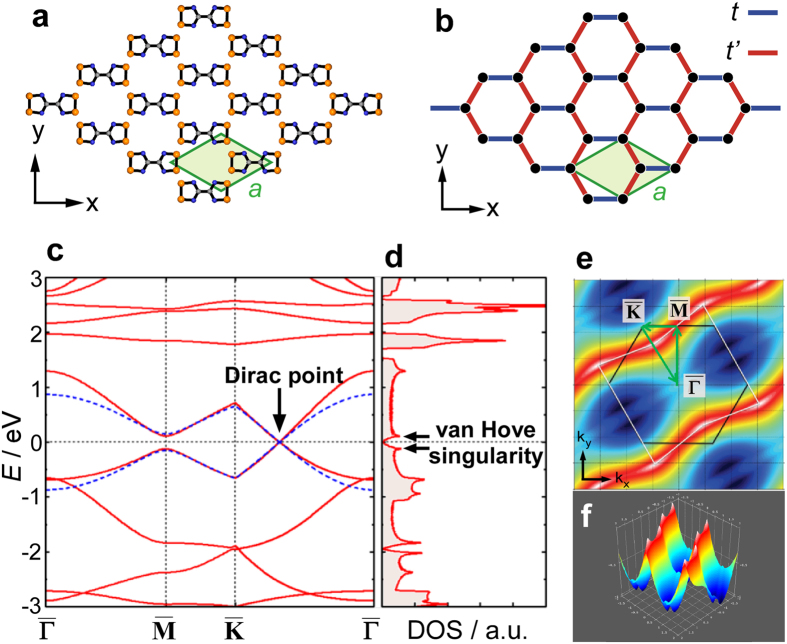
The DFT calculations of the BDTDA monolayer. (**a**) The BDTDA honeycomb lattice structure for DFT calculation. The lattice constant is fixed to the experimental value *a* = 7.65 Å. (**b**) A deformed graphene (tight-binding) model with intra-molecular coupling *t* and inter-molecular coupling *t*'. (**c**) Band structure for the BDTDA honeycomb monolayer (solid red lines). Dotted blue lines indicate the band structure obtained by the deformed graphene model with *t*=−150 meV and *t*' = 510 meV. (**d**) Corresponding density of states. The peaks at *E*=111 meV and −122 meV are attributed to the band edges of the 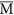-point. (**e**,**f**) Band map for the valence band.

The observed zero-bias anomaly in Fig. 3(a) can be explained by the presence of Dirac cones as discussed in the following. The honeycomb lattice of the BDTDA monolayer indicates that its electronic structure should be similar to that of a deformed graphene^24^; here two kinds of interactions, *t* and *t*′, are relevant parameters, namely the intra- and inter-molecular interactions between the unpaired electrons located at the 1,2,3,5-dithiadiazolyl pentagons (see Fig. 4(b)). If the strengths of the two interactions satisfy the relation *t* < 2*t*′, as expected from the short intermolecular distances, the Dirac cone which is a signature of honeycomb lattice^25^ can sustain the lattice deformation, only with change of the Brillouin zone shape from hexagonal to rectangular. Then the electronic density of states is reduced toward zero near the Dirac point, exhibiting a gapless structure^24^. In order to confirm this, we performed first principles calculations for the BDTDA honeycomb structure in Fig. 4(a) using the DFT package SIESTA^26^. Figure 4(c) shows the calculated band structure of an isolated BDTDA honeycomb single layer (solid red lines), in which a Dirac point is present on the 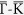 line. Of the calculated bands the dispersions of the two close to the Fermi level show good agreement with those obtained for a deformed graphene model^24^ with transfer integrals *t* = −150 meV and *t*′ = 510 meV (dotted blue line). This agreement suggests a strong intermolecular interaction between the pentagonal 1,2,3,5-dithiadiazolyl groups, and thus is consistent with the short intermolecular distance between the pentagon centers. The corresponding density of states shown in Fig. 4(d) exhibits a small dip at the Fermi energy as expected. In the actual sample, the electronic band structure shown in Fig. 4(c) may be smeared due to the finite interaction between the BDTDA molecules and the Cu substrate, but the dip structure can survive if the interaction is not too strong. We note that the observed zero bias anomaly might also be attributed to the anti-resonance of the Kondo effect originating from the Fano effect^27^,^28^,^29^,^30^, but this possibility is safely excluded. The result of our spin-polarized DFT calculation clearly shows that the BDTDA layer constitutes a spin singlet system as a whole, which stems from the strong intermolecular interaction *t*′. This indicates the absence of isolated local spins at individual molecules and makes emergence of the Kondo effect very unlikely.

It is certain that the BDTDA monolayer on Cu(111) will become a novel research target as a 2D electronic system in organic electronics and spintronics. Particularly, the possibility of realizing a molecular graphene through self-assembly process is interesting in terms of fabricating a new class of topological insulators^31^,^32^,^33^,^34^. We also envision creating a high-*T*_c_ superconductor through self-assembly of BDTDA monolayer on an insulating substrate. This is because the van Hove singularities are located near the Fermi level, *i.e.* at *E* = 111, −122 meV (see Fig. 4(d)). The resulting high density of states at these energies will increase the superconducting transition temperature substantially if the Fermi level can be tuned there chemically or electrostatically^35^. A high anisotropy of electron conduction is also expected judging from the DFT-calculated band dispersion, which is unique considering the structural uniformity of this molecular layer.

## Conclusion

We revealed that the thiazyl diradical, BDTDA, forms a well-ordered monolayer honeycomb lattice consisting of paramagnetic corners with unpaired electrons on Cu(111), due to its strong self-assembling force. This lattice is commensurate with the substrate, and is completely different from the bulk crystal structure. The STS measurements showed a characteristic zero-bias anomaly centered at the Fermi energy in the d*I*/d*V* spectra. The band structure calculation indicated that the origin of the zero-bias anomaly could be interpreted in terms of Dirac cones. The present study strongly suggests that the interface nanostructures of the organic radicals with strong self-assembling abilities will make exotic electronic/spin structures that are attractive from the viewpoints of both fundamental and applied sciences.

## Methods

### Preparation and Purification of BDTDA

The thiazyl diradical BDTDA was prepared according to the procedure described in the literature^11^,^12^ and purified by sublimation at 450 K under a reduced pressure of 1 Pa.

### Sample Preparation and STM Measurements

The sample preparations and the STM/S measurements were performed in an ultrahigh vacuum chamber equipped with a low-energy electron diffraction (LEED) system, an Auger electron spectroscopy (AES) apparatus, and an STM under a base pressure of <1 × 10^–8^ Pa. The Cu(111) substrates were cleaned by repeated Ar^+^-ion sputtering (1 keV) and annealing at 673 K. The tungsten tips used in these experiments were prepared by annealing and Ar^+^-ion bombardment. BDTDA was outgassed at 393 K for a few minutes, and then deposited on the clean surface at room temperature. The deposition rate was estimated as ~0.1 nm/min at 353 K by a quartz microbalance and the obtained STM images. After deposition, we annealed the samples at 323 K for more than 1 hour to enlarge the size of the BDTDA islands.

### Density Functional Theory Calculation

We perform first principles calculations using the density functional package SIESTA^26^ to investigate the electronic properties of free standing BDTDA monolayer. We consider generalized gradient approximation (GGA) and Perdew–Burke–Ernzerhof (PBE) exchange and correlation functional^36^ for spin polarized calculations with a double zeta polarized (DZP) basis set. Sufficient vacuum has been created in the non-periodic directions (*z*-axis) to avoid any interactions within adjacent unit cells. We consider 400 Ry energy cut-off for real space mesh size and a Brillouin zone sampling over 30 × 30 × 1 Monkhorst–Pack grid for the geometrical relaxation until the force on each atom reaches 0.04 eV/A. Note that, the lattice vectors are kept unrelaxed in their experimental values to get proper insight of the experimental observations. For precise determination of the density of states, we perform electronic structure calculations of the relaxed structure considering 5100 k-points with a k-point sampling over 100 × 100 × 1 Monkhorst–Pack grid. To locate the position of the Dirac points, we scan the two-dimensional Brillouin zone with very dense k-point mesh.

## Additional Information

**How to cite this article**: Yamamoto, M. *et al.* Self-assembled honeycomb lattice in the monolayer of cyclic thiazyl diradical BDTDA (=4,4'-bis(1,2,3,5-dithiadiazolyl)) on Cu(111) with a zero-bias tunneling spectra anomaly. *Sci. Rep.* **5**, 18359; doi: 10.1038/srep18359 (2015).

## Supplementary Material

Supplementary Information
