# Supplementary material for: Self-assembled honeycomb lattice in the monolayer of cyclic thiazyl diradical BDTDA (= 4,4′-bis(1,2,3,5-dithiadiazolyl)) on Cu(111) with a zero-bias tunneling spectra anomaly
Source: Sci Rep. 2015 Dec 17;5:18359. doi: 10.1038/srep18359 (PMC4683616; doi:10.1038/srep18359)
Supplement: Supplementary Information [file srep18359-s1.pdf]

Supplementary Information

Self-assembled honeycomb lattice in the monolayer of cyclic thiazyl diradical BDTDA (=4,4’-bis(1,2,3,5-dithiadiazolyl)) on Cu(111) with a zero-bias tunneling spectra anomaly

Masayuki Yamamoto, Rie Suizu, Sudipta Dutta, Puneet Mishra, Tomonobu Nakayama,

Kazuyuki Sakamoto, Katsunori Wakabayashi, Takashi Uchihashi, and Kunio Awaga

Contents

Figure S1. Single crystal structures of BDTDA S2

Figure S2. Bias voltage dependence of STM topographies S2

Figure S3. Measuring position dependence of d*I*/d*V* spectra S3

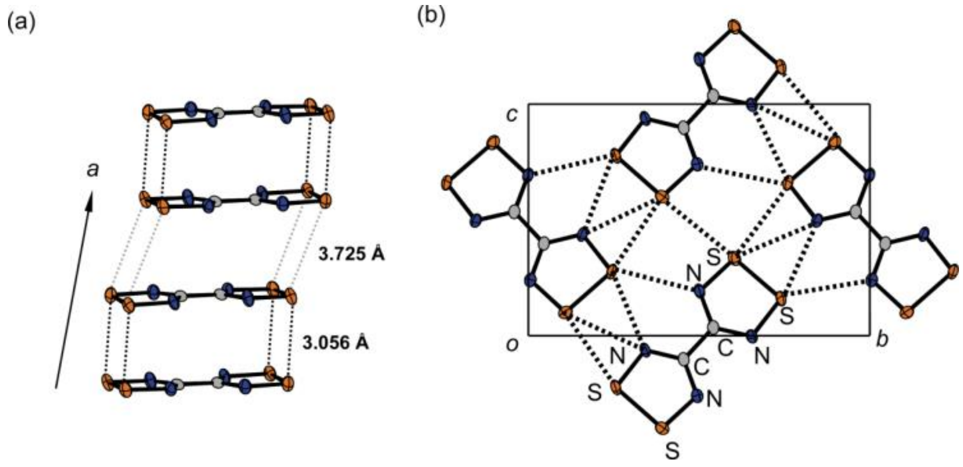

Figure S1. Single crystal structures of BDTDA at 173 K (a) stacking, (b) *bc* plane; Monoclinic, $P2_1/c$, a = 6.698(2) Å, b = 11.520(4) Å, c = 8.316(3) Å, b = 110.132(4) °, V = 602.5(4) Å^3, $R1/wR2$ = 0.0221/0.0660, GOF = 1.063.

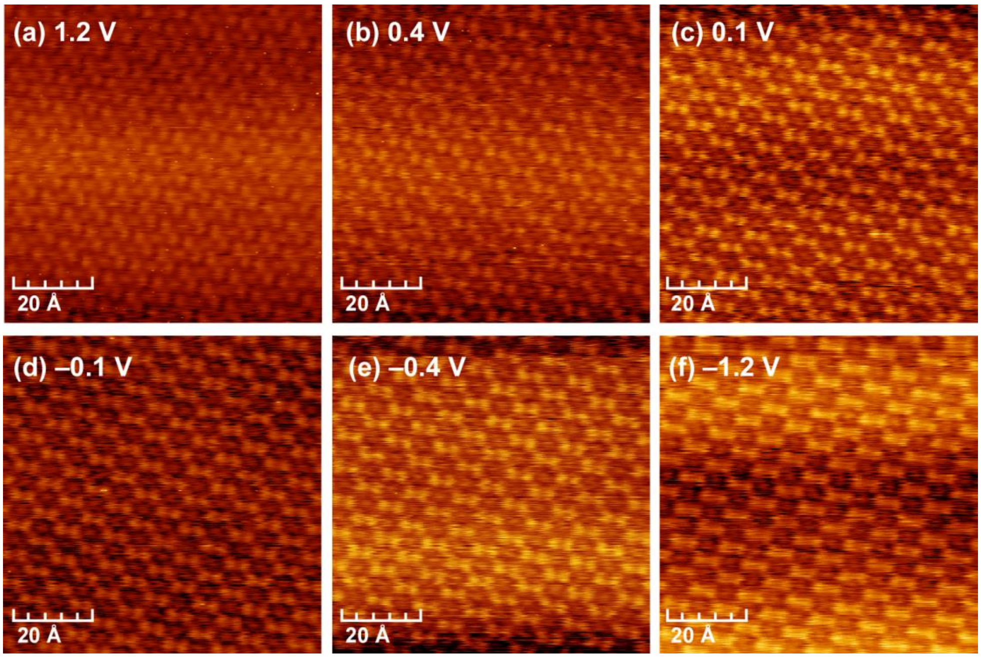

Figure S2. Bias voltage dependence of STM topographies.

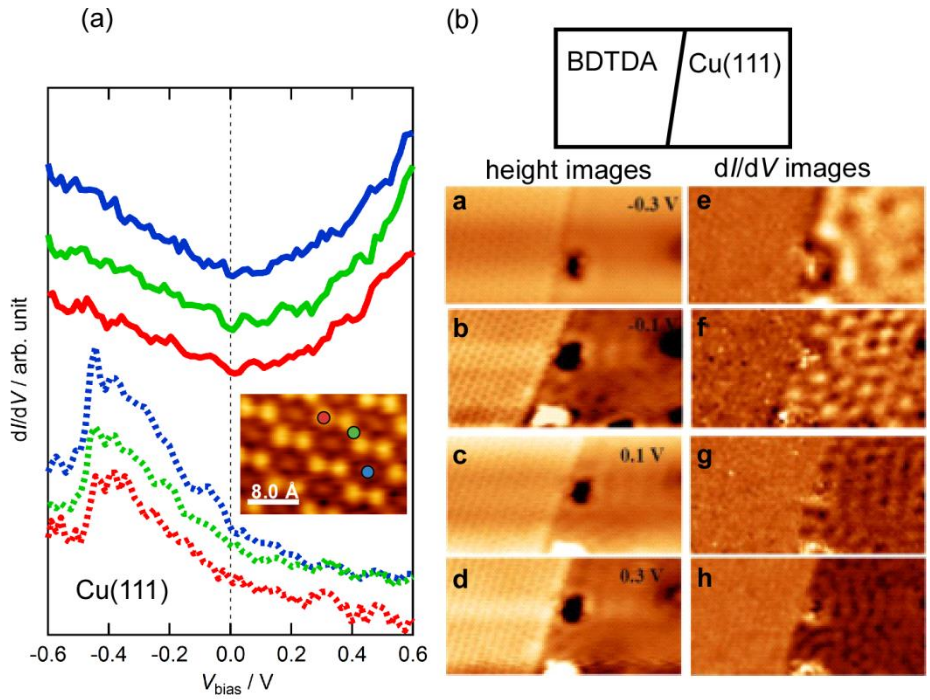

Figure S3. (a) Measuring position dependence of d*I*/d*V* spectra of BDTDA/Cu(111) on a thiazyl ring (red solid line), a node between rings (green solid line) and a center of honeycomb superlattice (blue solid line), and clean Cu(111) substrate just after measuring the corresponding positions of BDTDA honeycomb superlattice. The spectra are offset for clarity. (b) **a**-**d**; Height images, **e**-**h**; d*I*/d*V* images of BDTDA/Cu(111). V_{bias} = -0.3 V (**a** and **e**), -0.1 V (**b** and **f**), 0.1 V (**c** and **g**), and 0.3 V (**d** and **h**). These d*I*/d*V* images clearly indicate the presence of the standing wave on Cu(111).
